# Photolysis of Caged-GABA Rapidly Terminates Seizures *In Vivo*: Concentration and Light Intensity Dependence

**DOI:** 10.3389/fneur.2017.00215

**Published:** 2017-05-18

**Authors:** Dan Wang, Zhixin Yu, Jiaqing Yan, Fenqin Xue, Guoping Ren, Chenxi Jiang, Weimin Wang, Yueshan Piao, Xiaofeng Yang

**Affiliations:** ^1^Neuroelectrophysiological Laboratory, Xuanwu Hospital, Capital Medical University, Beijing, China; ^2^Center of Epilepsy, Beijing Institute for Brain Disorders, Beijing, China; ^3^Center of Epilepsy, Center for Brain Disorders Research, Capital Medical University, Beijing, China; ^4^College of Electrical and Control Engineering, North China University of Technology, Beijing, China; ^5^Core Facilities Center, Capital Medical University, Beijing, China; ^6^Department of Pathology, Xuanwu Hospital, Capital Medical University, Beijing, China

**Keywords:** focal neocortical epilepsy, 4-aminopyridine, seizure, photolysis, RuBi-GABA

## Abstract

The therapy of focal epilepsy remains unsatisfactory for as many as 25% of patients. The photolysis of caged-γ-aminobutyric acid (caged-GABA) represents a novel and alternative option for the treatment of intractable epilepsy. Our previous experimental results have demonstrated that the use of blue light produced by light-emitting diode to uncage ruthenium-bipyridine-triphenylphosphine-c-GABA (RuBi-GABA) can rapidly terminate paroxysmal seizure activity both *in vitro* and *in vivo*. However, the optimal concentration of RuBi-GABA, and the intensity of illumination to abort seizures, remains unknown. The aim of this study was to explore the optimal anti-seizure effects of RuBi-GABA by using implantable fibers to introduce blue light into the neocortex of a 4-aminopyridine-induced acute seizure model in rats. We then investigated the effects of different combinations of RuBi-GABA concentrations and light intensity upon seizure. Our results show that the anti-seizure effect of RuBi-GABA has obvious concentration and light intensity dependence. This is the first example of using an implantable device for the photolysis of RuBi-GABA in the therapy of neocortical seizure, and an optimal combination of RuBi-GABA concentration and light intensity was explored. These results provide important experimental data for future clinical translational studies.

## Introduction

Epilepsy affects approximately 50 million people worldwide (1). Even with optimal drug treatment, one quarter of epileptics have seizures which are inadequately controlled by even maximal doses of medications. This subset of epileptic patients is defined as intractable (2). Although surgery has clearly benefited some patients with intractable epilepsy, it has proved to be less successful in focal neocortical epilepsy because proximity to the eloquent cortex prevents or restricts the extent of resection (3, 4). Some alternative therapies have been shown to have the potential to control seizures, such as vagus nerve stimulation, responsive cortical stimulation, deep brain stimulation (DBS), transcranial magnetic stimulation, transcranial direct current stimulation, local delivery of drugs, focal cooling, and chemogenetic and ketogenic diets (5–14). However, all of these possible therapies have had either limited success or have yet to be tested in patients. Many patients continue to have poorly controlled epilepsy, with significant effects upon their health and welfare (15, 16).

Over the past two decades, advances in optical technology have shown that we may be able to exploit optical technology to control the excitability of neurons (17–21). Furthermore, optical techniques are able to control neuronal excitability with precise temporal and spatial resolution; consequently, this technique has been shown to be highly attractive for controlling epilepsy. Several groups have reported that optogenetic approaches successfully attenuated epileptiform activity in different rodent models of epilepsy. There have been many reports which have successfully described the control of seizure activity by using the optogenetics method in different rodent epilepsy models (22–26). Unfortunately, optogenetic technology requires permanent alteration of the genome in transfected neurons and appears to modify their fundamental neurobiology. This factor may affect the clinical translation of this method.

Compared with optogenetics, caged compounds, including caged-γ-aminobutyric acid (GABA), may provide a more practical approach for exploiting the potential of optical techniques to control pathological neuronal excitability, especially for the control of epilepsy. Caged compounds contain a photolabile protecting group which can be removed by exposure to light, thus liberating a bioactive compound which can control neuronal excitability. The bioactive compounds used are usually natural proteins or neurotransmitters and are quickly taken up by neurons or glial cells, meaning that caged compounds do not change the internal environment. There are some studies that have already proven that uncaging caged-GABA can control neuronal excitability (27, 28).

Our previous experiments have already demonstrated that the photolysis of caged-GABA, 4-[([2H-benzopyran-2-one-7-amino-4-methoxy] carbonyl) amino] butanoic acid (BC204), by a small, ultraviolet light-emitting diode (UV LED), can terminate “ictal-like” events in cultured neurons and in rat hippocampal slices (17, 29). We also explored the use of a small LED to generate blue light to uncage ruthenium-bipyridine-triphenylphosphine-c-GABA (RuBi-GABA) to control the seizure activity in both brain slices and *in vivo* (30). Since this previous *in vivo* experiment was just for proof of principle, the LED we used could not be implanted *in vivo*, and we did not explore the optimal caged-GABA concentration and light intensity to control seizures. The current study was designed to promote future clinical translational studies. We use an implantable fiber to introduce a blue laser into the neocortex to cause photolysis of RuBi-GABA and explore the optimal RuBi-GABA concentration and blue laser intensity necessary for the termination of neocortical seizures in a rat model.

## Materials and Methods

### Animals and Chemicals

Adult male Sprague-Dawley rats weighing 250–350 g were used in our experiment and were acquired from the animal facility of Capital Medical University. Animal care and protocols were approved by the Ethics Committee on Animals Care and Usage of Capital Medical University. RuBi-GABA was purchased from Abcam Biochemicals (Cambridge, UK). Isoflurane was purchased from Ruiwode (Shenzhen, China), and all other chemicals were purchased from Sigma-Aldrich (St Louis, MO, USA).

### Neocortical Seizure Model and Electroencephalography

Rats were anesthetized with isoflurane (2%) and then placed on a Stoelting stereotaxic frame (Stoelting, Wood Dale, IL, USA). A 4-mm diameter craniotomy was performed over the anterior left hemisphere using a dental drill. During drilling, the skull was continuously irrigated with artificial cerebrospinal fluid (ACSF) to prevent the underlying brain from overheating. After the craniotomy was performed, we created a reservoir around the cranial window using dental cement which was then filled with RuBi-GABA solutions. A 2-mm slit was then placed in the dura which allowed the RuBi-GABA that filled the reservoir to directly diffuse into the neocortical surface.

**Figure 1:**
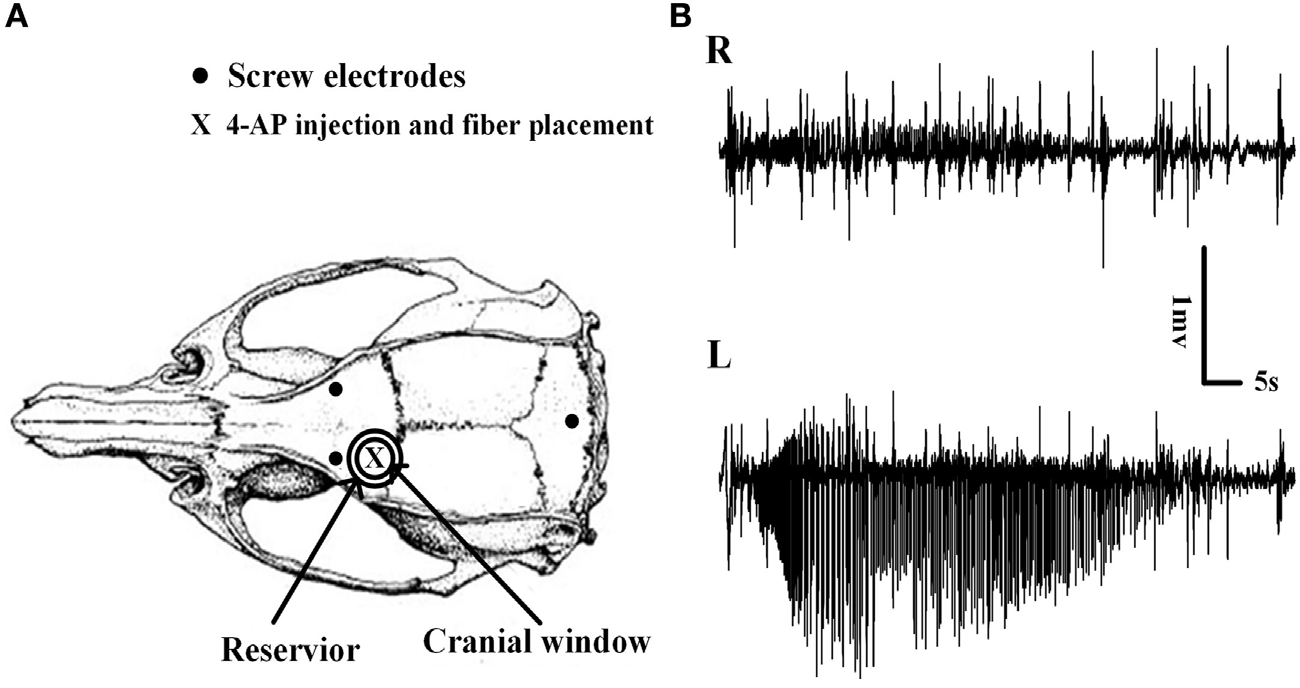
**Schematic diagram of the craniotomy, electrodes, and the placement of implantable optical fibers**. **(A)** Cranial window, electroencephalogram electrodes, and implantable optical fiber placement. The cranial window overlaid the left motor cortex, and the reservoir around the cranial window was filled with RuBi-GABA solutions. The site of the 4-aminopyridine (4-AP) injection and the optical fiber placement are shown. **(B)** Examples of 4-AP-induced neocortical seizures. Seizures were recorded only on the 4-AP injection side which thus demonstrated the existence of focal seizure.

Two screw electrodes were symmetrically placed in the skull over each hemisphere to differentially record the electroencephalogram (EEG) between each hemisphere. The reference electrode and ground electrode were implanted in the neck muscles (Figure 1A). EEGs were recorded using a Cerebus Neural Signal Processing System (Blackrock, Salt Lake City, UT, USA) at a sampling rate of 2000 Hz, and all the data were stored on a personal computer. We commenced EEG recording before RuBi-GABA pretreatment and continued to record throughout the entire experiment. Electrophysiological data were analyzed offline using the Matlab program (Math Works, Natick, MA, USA).

When we finished the surgery, we added 200 µl of ACSF or RuBi-GABA solution (0.1 or 0.2 mM in ACSF) to the reservoir, respectively. After 45 min of pretreatment, we induced focal neocortical seizures by injecting 0.5 µl of a 4-aminopyridine (4-AP) solution (25 mM in ACSF) into the neocortex at a depth of 0.5 mm, at a position 2 mm anterior to the bregma and 2.5 mm from the midline, using a commercial oocyte injection system (Drummond Scientific, Broomall, PA, USA) coupled to a glass micropipette (tip diameter approximately 100 µm). The injection was carried out over a 5-min period to minimize cortical damage, and the pipette was maintained for an additional 5 min to minimize leakage of the 4-AP.

All ictal and interictal durations of seizures in our experiment were independently and blindly analyzed offline by two experienced reviewers. We observed close agreement between the two reviewers. Because we need a few seconds to confirm the seizure onset before we start the photolysis process, so there is a lantency between seizure onset to photolysis process was started. In order to accurately show the antiepileptic effects of RuBi-GABA, so we also corrected seizure durations by subtracting the latency in the amount of time from seizures onset to the initiation of photolysis process. Owing to high time resolution, wavelet analysis was applied to depict time frequency spectrum to reflect dynamic change of seizures during uncaging.

To observe the change in EEG power and achieve more accurate results, the fast Fourier transform (FFT)-based power spectral analysis method with excellent frequency resolution was used to calculate the power of each frequency band activity in seizures. Power spectral histograms were calculated using 4000 points Hamming window with 2000 points overlap and 4000 FFT points. The EEG frequency was decomposed into several frequency activity: delta frequency band (1–4 Hz), theta frequency band (4–8 Hz), alpha frequency band (8–13 Hz), and beta frequency band (13–30 Hz). Because of the great individual variability in the power of EEG in the rats, these variations will directly affect the results of the analysis. Therefore, we normalized the power of EEG in different frequency bands for each rat. The normalized approach is the power of seizure with photolysis of RuBi-GABA divided by the mean of the power of seizures without photolysis in the rats.

To observe the change of EEG power from baseline to seizures, we randomly chose two baseline EEG segments prior to the injection of 4-AP from each rat, with a duration equal to the mean duration of seizures in the control group (CG). Then, the powers of these EEGs were calculated using the FFT and compared between baseline and CGs.

### Photolysis of Caged-GABA

We used an implantable optical fiber (0.39NA, 200 µm diameter; Doric Lenses Inc, Quebec, Canada) terminated in 1.25-mm ceramic ferrules (5 mm length; Ruiwode, Shenzhen, China) to introduce a blue laser, produced by a high-power laser driver (473 nm; SLOC, Shanghai, China), into the neocortex (4-AP injection site) to cause the photolysis of RuBi-GABA. The optical power delivered at the fiber tip was calibrated with an optical power meter (PM121D; Thorlabs, Newtown, NJ, USA) before experiments. When seizure onset was confirmed, we manually triggered the laser driver to start the photolysis process for 30 s. To avoid the long-lasting effects of the photolysis of RuBi-GABA upon subsequent experimental results, we did not trigger the next photolysis activity until the seizure duration had completely recovered to control levels. In addition, to assure sufficient RuBi-GABA for each photolysis process and avoid the effect of GABA which released from the solution in the reservoir on the subsequent experiments, we replaced the solution in the reservoir after each photolysis activities.

Animals were divided into four different groups: a CG (*n* = 8), an illumination-only group (IC, *n* = 6), a 0.2-mM RuBi-GABA without illumination group (RC, *n* = 6), and a RuBi-GABA with illumination group (RI, *n* = 24). The RI group was further divided into four sub-groups (*n* = 6 for each group): a 0.1-mM RuBi-GABA with 15-mW photolysis group (RI_1_), a 20-mW photolysis group (RI_2_), a 0.2-mM RuBi-GABA using 10-mW photolysis (RI_3_), and a 15-mW photolysis (RI_4_) group. All experiments involving RuBi-GABA were carried out in a darkened room with only occasional red lamp illumination.

### Histology

All rats were sacrificed 3 days after the acute experiments with an overdose of isoflurane (5%). Brain tissue was removed immediately and preserved in 10% formalin. After fixation, the tissue was embedded in paraffin. Coronal 4-µm sections were cut in a manner which included the area around the injection site and the corresponding contralateral neocortex. Sections were then examined with hematoxylin/eosin for signs of necrotic injury, TdT-mediated dUTP-biotin nick-end labeling (TUNEL) staining for evidence of apoptotic death, and glial fibrillary acidic protein (GFAP) staining to evaluate possible glial proliferation.

### Statistics Analysis

All data are presented as mean ± SEM. Data were analyzed using SPSS 19.0 statistics software (IBM, Armonk, NY, USA). Seizure duration and the changes of EEG power among different groups were analyzed by analysis of variance. The *post hoc* test used least significant difference. The Wilcoxon signed-rank test was used to compare non-normally distributed data (interictal time). A *P*-value of <0.05 was considered to be statistically significant.

## Results

### Effect of RuBi-GABA Uncaging upon *In Vivo* Seizures in a Concentration- and Light-Intensity-Dependent Manner

**Figure 2:**
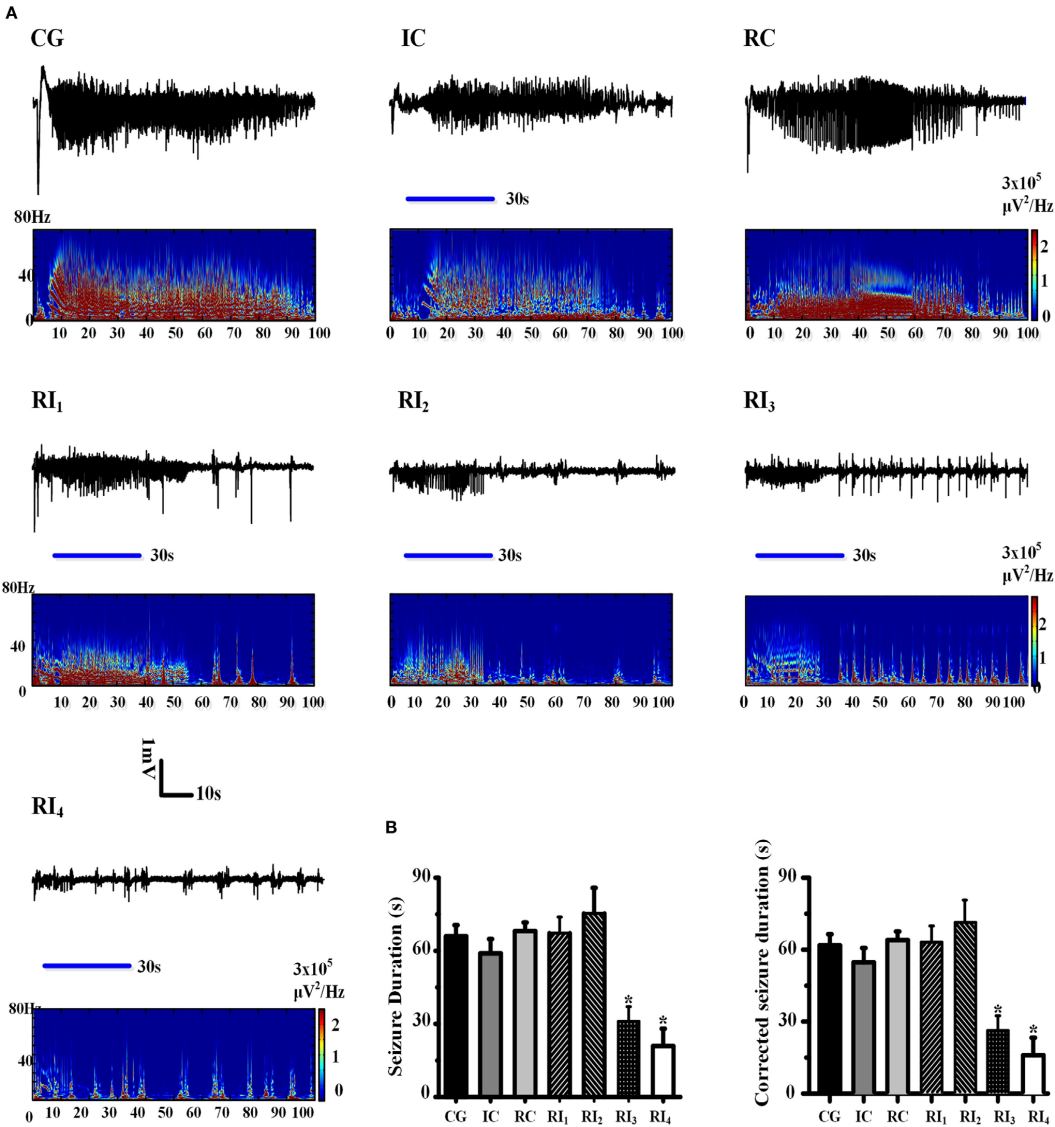
**Uncaging of ruthenium-bipyridine-triphenylphosphine-c-aminobutyric acid reduced 4-aminopyridine (4-AP)-induced neocortical seizures**. **(A)** The top panel of each group shows the electroencephalogram (EEG) record which demonstrates the seizure duration for each group. Morlet-wavelet EEG spectra from the seizures which were induced by 4-AP for each group are shown. The blue horizontal line shows the beginning and end points of the period of illumination. **(B)** Comparison of seizure durations among experimental groups. The duration of seizures was significantly shorter in the RI_3_ and RI_4_ groups compared with the control group (*P < 0.05).

We did not detect any seizures following the surgery and the application of ACSF containing RuBi-GABA upon the brain surface. However, within 10 ± 1.23 min of 4-AP injection, animals developed recurrent electrographic focal seizures with a mean seizure duration and interictal period of 65.92 ± 4.60 and 263.90 ± 23.00 s, respectively; these conditions remained for 1.5 h (Figures 1B and 2A,B).

To explore whether the anti-seizure effects of RuBi-GABA were related to RuBi-GABA concentration and the intensity of illumination, we measured different combinations of RuBi-GABA concentrations and light intensities. We found that the seizure durations of these groups were significantly different [*F* (1, 6) = 2.337, *P* = 0.047]. Then, we tested the combination of 0.1-mM RuBi-GABA with 15 and 20 mW of illumination (30 s). Under these conditions, seizure duration was 67.25 ± 3.57 and 75.33 ± 10.48 s, respectively. Compared with the CG, there was no statistically significant difference (*P* = 0.905 and *P* = 0.585). However, when the concentration of RuBi-GABA was increased to 0.2 with 10 and 15 mW of light intensity, the seizure duration was significantly reduced to 31.00 ± 6.05 and 20.86 ± 7.20 s, respectively. Compared with the CG, these reductions were statistically significant (*P* = 0.047 and *P* = 0.017 vs. CG, Figures 2A,B).

Because manual confirmation of seizure onset requires a few seconds, there was a latency from the onset of seizure to the initiation of photolysis. These latencies in the IC group and the RI_1–4_ groups were 5.30 ± 0.96, 5.60 ± 0.45, 5.56 ± 0.41, 7.75 ± 2.21, and 4.56 ± 0.63 s, with an average latency of 5.69 ± 0.44 s.

To more accurately show the anti-seizure effect of the photolysis of RuBi-GABA, we corrected the duration of the seizures by subtracting the average latency (5.69 ± 0.44 s) from all seizure durations. There was also significant difference existed between these groups in the corrected seizure duration [*F* (1, 6) = 2.337, *P* = 0.047]. The duration of seizure after correction of the RI_1_ and RI_2_ groups was 61.56 ± 3.57 and 69.64 ± 10.48 s, respectively. Compared with the correct CG (60.23 ± 4.60 s), there was no statistically significant difference (*P* = 0.905 and *P* = 0.585). The corrected seizure duration of the RI_3_ and RI_4_ groups was 25.31 ± 6.05 and 15.13 ± 7.20 s, respectively. Compared with the corrected CG, these reductions were statistically significant (*P* = 0.047 and *P* = 0.017 vs. CG, Figures 2A,B).

**Figure 3:**
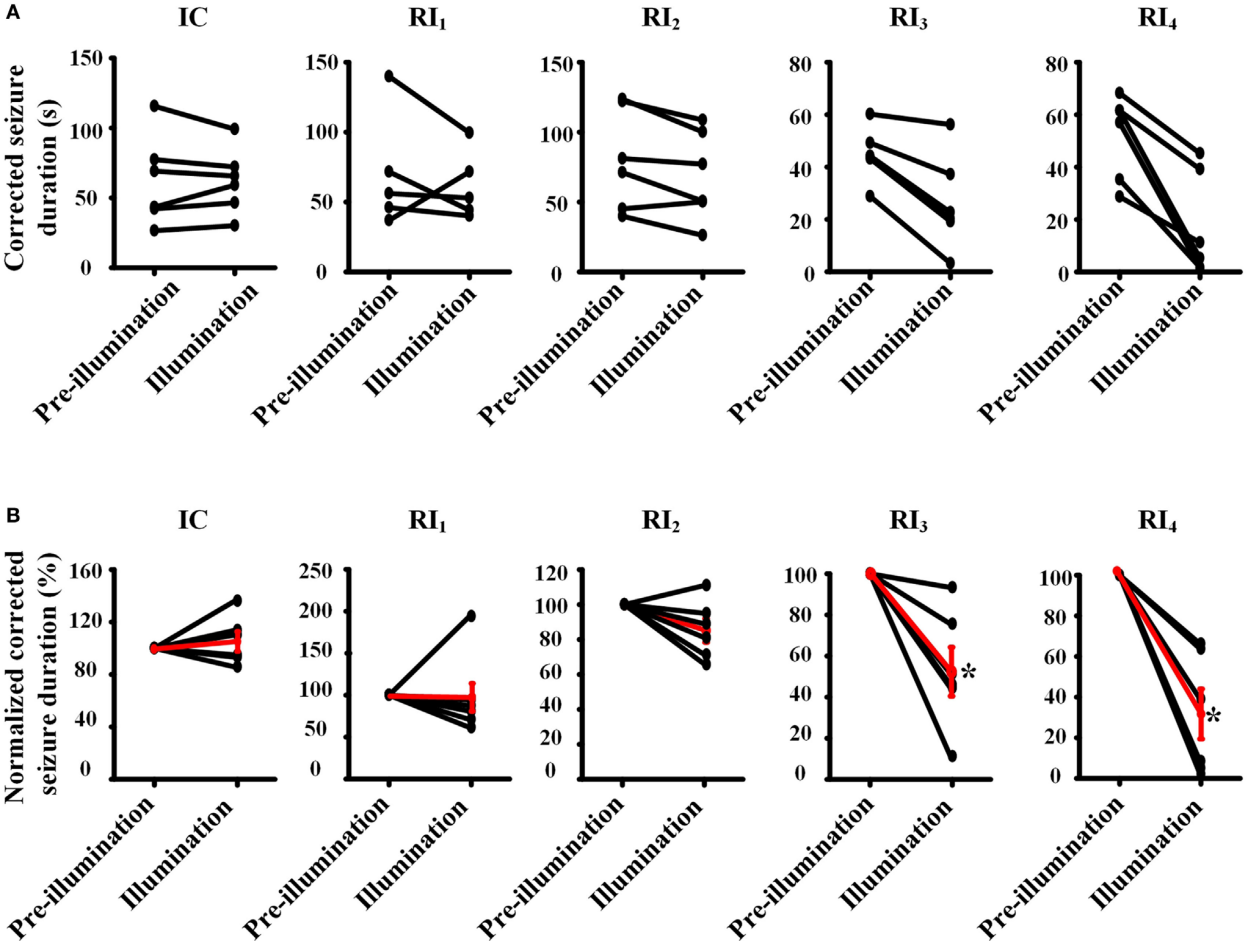
**Pair-wise comparison of seizure durations between pre-illumination and illumination on 4-aminopyridine-induced seizure in rats**. **(A)** Pair-wise comparisons of the duration of seizure between before and after illumination were performed for each animal (absolute time). **(B)** Normalized pair-wise comparisons of the duration of seizures between before and after illumination. The black line shows the comparison result for each animal, and the red line shows mean results for each group. Both results showed that the seizure durations are significantly decreased in the groups of RI_3_ and RI_4_.

To avoid the influence of long-lasting effect of RuBi-GABA photolysis to our experimental results, we also compared the corrected duration of each seizure with photolysis of RuBi-GABA and the previous seizure without photolysis process. Furthermore, we calculated the ratio of the corrected duration of seizures with and without photolysis process (normalized corrected seizure duration). Comparison of illumination or non-illumination, we did not find a significant reduction of seizure duration in IC, RI_1_, and RI_2_ groups, however, we found that illumination significantly shortened the duration of seizure in the RI_3_ and RI_4_ groups (Figure 3).

### The Long-lasting Effect of the Photolysis of RuBi-GABA to Control Seizure

**Figure 4:**
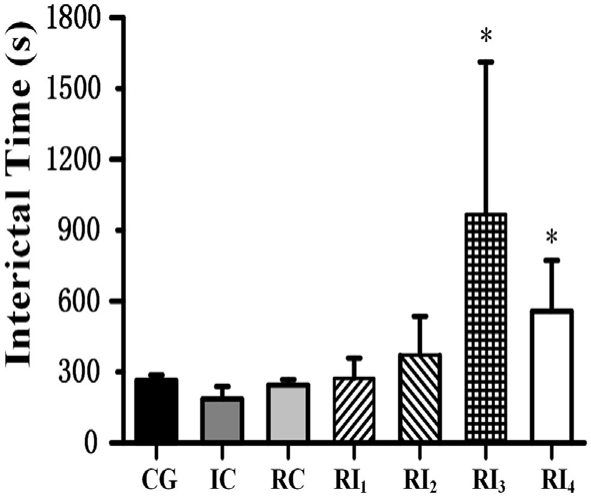
**Photolysis of ruthenium-bipyridine-triphenylphosphine-c-aminobutyric acid significantly extended interictal time**. The interictal time of group RI_3_ and group RI_4_ significantly increased compared with the control group (*P < 0.05). Data are presented as mean ± SEM.

When we tested the ability of GABA photolysis to control seizures, we found a phenomenon in which RuBi-GABA uncaging can not only quickly terminate seizures but also significantly prolong the intervals between seizures. When we used 0.2 with 10 and 15 mW of illumination, the interictal interval was significantly prolonged from 263.90 ± 23.00 s in the CG to 966.00 ± 646.00 and 556.75 ± 215.83 s, respectively (*P* < 0.05, Figure 4). Of particular note was that five of the 12 rats in the two experimental groups were seizure free following the first illumination. However, when we applied a combination of 0.1 mM RuBi-GABA and 15 and 20 mW of illumination, the interictal interval was 273.00 ± 85.31 and 370.78 ± 164.72 s, respectively, which was not significantly longer than the CG (*P* > 0.05).

### Uncaging of RuBi-GABA Alters the Power Spectrum of Neocortical Seizures

**Figure 5:**
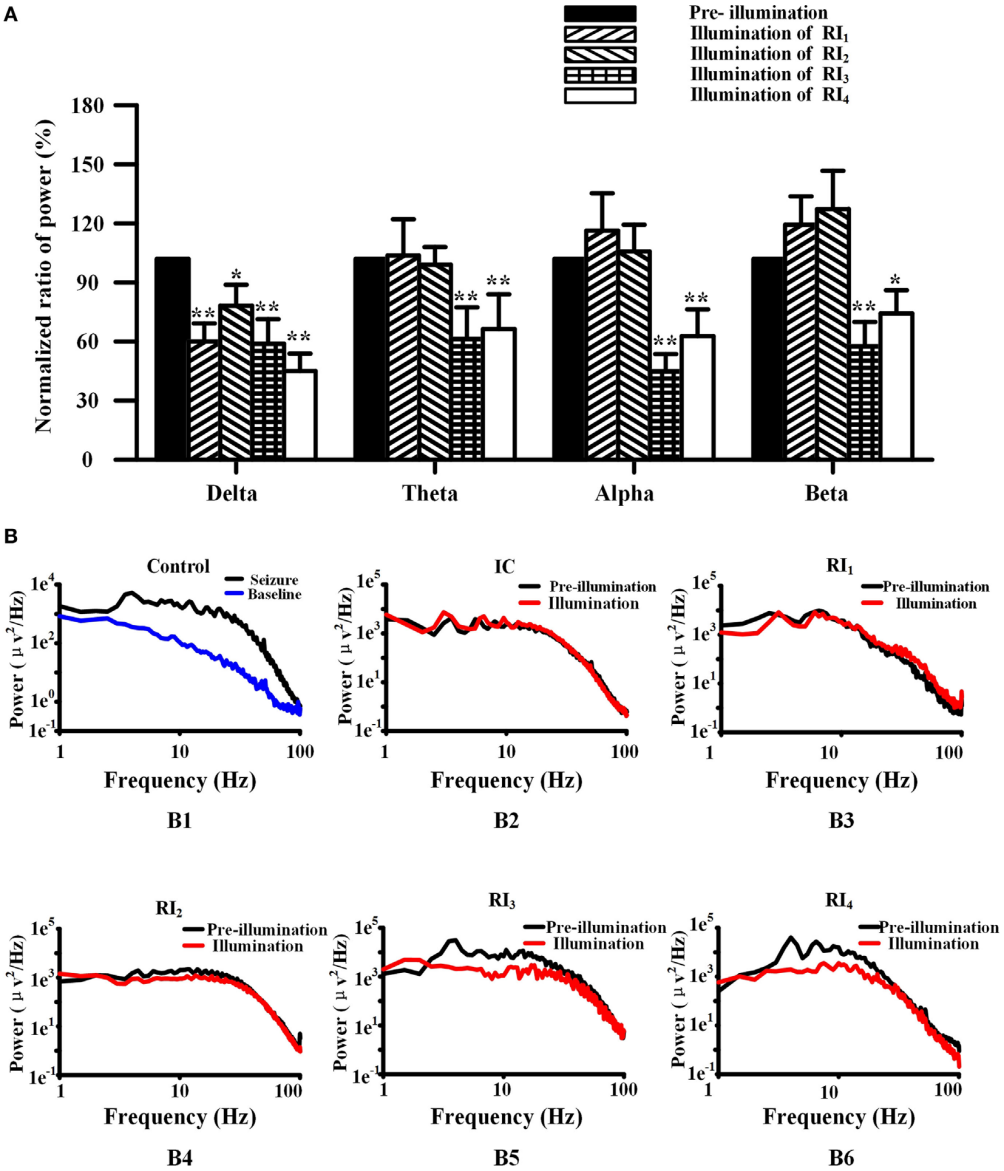
**Photolysis of ruthenium-bipyridine-triphenylphosphine-c-aminobutyric acid altered power in the frequency spectrum of neocortical seizures**. **(A)** The power of spectra during neocortical seizures was compared among all groups (normalized results). In the RI_1_ and RI_2_ groups, only the power of the delta band was reduced. However, the power of all frequency bands in the RI_3_ and RI_4_ groups were significantly reduced (*P < 0.05, **P < 0.01 *vs*. pre-illumination). **(B)** (B1) Power was calculated by the fast Fourier transform method of control seizures (black) and pre-seizure baseline (blue), showing the relatively high power of 4–7 Hz. **(B)** (B3-6) The power of seizures in the RI_1–4_ groups. The frequency peak in the 4–7 Hz band was shown in pre-illumination (black) for all groups and illumination in the RI_1–2_ groups, but was not present during illumination (red) in the RI_3–4_ groups.

We used FFT to calculate the spectral power of the EEG at baseline and control seizures, and seizures that treated by RuBi-GABA uncaging. Compared with baseline, the seizure significantly increases in the spectral power of delta, theta, alpha, and beta frequency activity (Figure 5B). In particular, we found that large spectral peaks were present at approximately 4–7 Hz during the control seizures. When we tested the combination of 0.1 mM RuBi-GABA with 15 and 20 mW of illumination (30 s), only the power of delta during the seizures was significantly reduced compared with controls, but the power of other frequency bands did not change significantly (*P* < 0.05 *vs*. pre-illumination). However, in the groups receiving 0.2-mM RuBi-GABA with 15 and 20 mW of illumination (30 s), we found that the power in all frequency bands was significantly reduced compared with the CG (*P* < 0.05 vs. pre-illumination, Figure 5A). In particular, the large spectral peaks, which were present in the control seizure animals at approximately 4–7 Hz, disappeared (Figure 5B).

### Uncaging of RuBi-GABA Did Not Produce Neuropathological Changes

**Figure 6:**
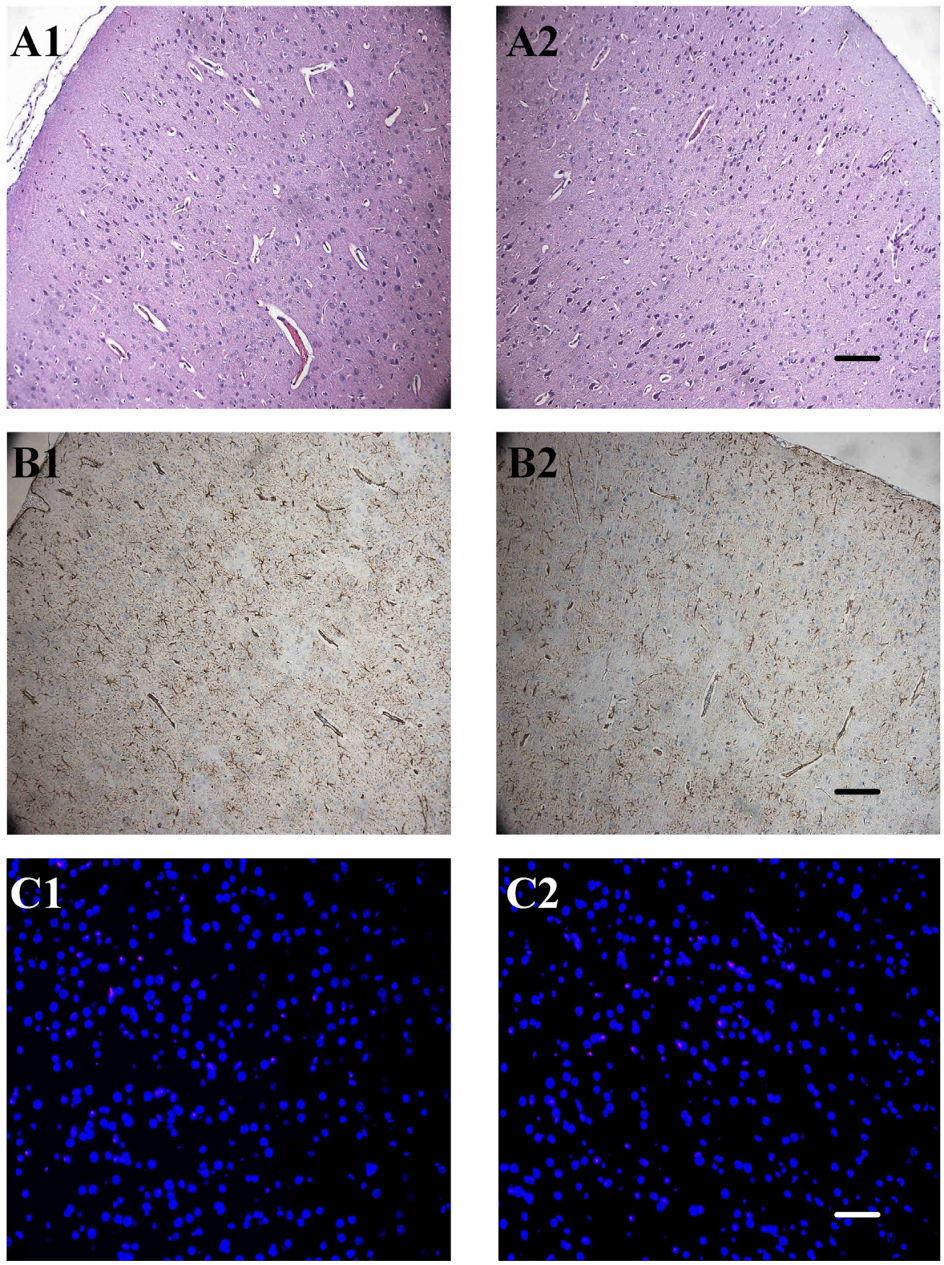
**The effect of ruthenium-bipyridine-triphenylphosphine-c-aminobutyric acid uncaging upon neocortical neurons and glia**. **(A)** Hematoxylin/eosin staining revealed no detectable differences between the control neocortex (A1) and the contralateral neocortex which was exposed to photolysis during the RuBi-GABA process (A2, 0.2-mM RuBi-GABA with 15 mW of illumination). **(B)** Glial fibrillary acidic protein (GFAP) staining of the control cortex (B1) showed no significant difference with the photolysis side (B2). **(C)** Section of control group cortex (A1, B1, and C1) and group RI_4_ cortex (A2, B2, and C2). A: Hematoxylin/eosin staining, B: GFAP staining, C: TdT-mediated dUTP-biotin nick-end labeling (TUNEL) staining. Scale, A2 and B2: 100 µm, C2: 50 µm.

We were concerned that the products produced by uncaging of the RuBi-GABA, and the phototoxicity experienced during the photolysis process, may damage the underlying neocortex. Therefore, we performed routine hematoxylin/eosin staining, GFAP staining, and TUNEL staining 3 days after seizures to examine neuronal loss, neuronal necrosis, apoptotic death, and glial proliferation. The hematoxylin/eosin staining results showed that 4-AP-injected/RuBi-GABA-treated neocortex was completely indistinguishable from controls. We did not find any neuronal necrosis or loss. Furthermore, we did not detect TUNEL-positive cells in 4-AP-injected/RuBi-GABA-treated neocortex or the corresponding contralateral neocortex by TUNEL staining. Examination of GFAP-stained sections showed some sporadic astrocytes in the 4-AP-injected and RuBi-GABA-treated neocortex which were not detected in the control neocortex (Figure 6).

## Discussion

Previous research on rat brain slices from our laboratory has already demonstrated that we can use a small LED to directly induce the photolysis of caged-GABA to terminate focal seizures (17, 29, 30). Because these earlier reports merely represented an important proof of principle, we did not utilize an implantable light source. Furthermore, there is a distinct lack of systematic experimental studies on the effects of caged-GABA concentration and light intensity using *in vivo* seizure models. In our previous experiments, the light source was a small LED positioned 3–4 mm above the brain surface so that the intensity of light entering the neocortex was significantly reduced. In this current study, we used an implantable optical fiber to introduce blue laser light into the neocortex on a 4-AP-induced acute seizure rat model and investigated the effects of different combinations of RuBi-GABA concentrations and light intensity upon seizure. To our knowledge, this is the first study to use an implantable optical fiber to uncage caged-GABA *in vivo* to control seizures. We also explored the optimal combination of RuBi-GABA concentration and light intensity for seizure control.

Our results showed that laser photolysis of RuBi-GABA by an implantable optical fiber not only rapidly terminated seizures but also prevented seizures in a model of focal neocortical seizures. In addition, we also found that the control of seizures by the photolysis of GABA showed dependence upon RuBi-GABA concentration and light intensity.

We need to emphasize that the uncaging of RuBi-GABA can successfully stop 4-AP-induced seizures, which are very intense focal seizures and cannot be stopped by doses of intraperitoneal diazepam, a technique known to terminate other types of rat status epilepticus (17, 31). In view of the local severity of the model employed in this study, RuBi-GABA uncaging may have a more effective anti-seizure effect in more realistic seizure models or clinical epilepsy. Furthermore, we may be able to reduce the concentration of RuBi-GABA or light intensity for *in vivo* seizures, which are milder and less resistant to therapy.

In our experiments, we found that the power of delta, theta, alpha, and beta frequency activity of EEG during seizures was significantly increased compared with baseline. In particular, a large spectral peak at around 4–7 Hz was present during control seizures. When we utilized a low RuBi-GABA concentration (0.1 mM) with 15- and 20-mW illuminations, only the power of delta was significantly reduced compared with control seizures and the large spectral peaks at around 4–7 Hz were still present. In this case, the duration of seizure was not reduced. However, when we increased the RuBi-GABA concentration to 0.2 with 10 and 15 mW of illumination, not only were all EEG bands suppressed but also the large spectral peaks at around 4–7 Hz (the theta band) disappeared. These results suggest that the photolysis of RuBi-GABA can suppress spectral band power in a concentration- and light-intensity-dependent manner. Previous literature demonstrated that theta bands are more likely to be generated from deep cortical layers and can only be inhibited using stronger methods (32).

There has been a significant amount of research pertaining to the relationship between theta waves and the generation of seizures. One previous study indicated that increased magnetoencephalogram connectivity of theta bands between different brain areas was related to a higher total number of seizures in operated glioma patients suffering from epileptic seizures (33). Another study, carried out in pilocarpine-induced rat epilepsy models, reported that much of the increased pre-ictal firing of neurons in the subiculum and CA1 correlated with pre-ictal theta activity (34). Sedigh-Sarvestani and colleagues also reported a study using a rat ventral hippocampus-injected tetanus toxin epilepsy model and stated that rapid eye movement sleep and exploratory wake, both of which expressed prominent hippocampal theta rhythm, preceded 81% of all seizures (35). Moreover, the use of DBS in the treatment of resistant epilepsy could also inhibit theta band power, which may represent a potential mechanism of DBS action for the treatment of epilepsy (36).

Over the past decade, several groups have reported that optogenetic approaches have successfully attenuated epileptiform activity in rodent models of epilepsy, thus providing proof of principle that this approach may be effective in controlling epilepsy (23, 25, 37, 38). Unfortunately, unanswered questions abound, and many of the fundamental questions have to be addressed before optogenetic therapeutic techniques become a reality (39). First, optogenetic therapeutics will require the delivery of genetic constructs into the body by means of a viral vector, but this technology is far from fully established in humans. Second, optogenetic technology requires introducing exogenous receptors or ion pumps into membrane of human neurons that may alter other fundamental cellular processes (26). Furthermore, transfection will irreversibly modify the genome of post-mitotic neurons. It will, therefore, take years to determine whether this technology will have a place in human epilepsy therapy.

One important issue to consider is whether the photolysis of RuBi-GABA would cause damage to the neocortex in future clinical translation research. In our study, we performed pathological and immunohistochemical examinations to detect whether the uncaging of RuBi-GABA causes damage to neurons and glial cells. We found no evidence of damage to neurons and glial cells, such as necrosis or apoptosis. Although there was a mild glial proliferation following the treatment by RuBi-GABA uncaging, we believe that this was due to the physical stimulation of the neocortex by craniotomy and fiber implantation. Based on these results, we are confident that the photolysis of RuBi-GABA to control seizures represents a safe and feasible technique for clinical practice.

Our results represent a very important step in developing this novel alternative therapeutic method toward clinical application and provide vital parameters with which to design implantable optical devices for epilepsy patients. The favorable anti-seizure effects and relative safety of this method suggest that RuBi-GABA uncaging has the potential to be a minimally invasive, very specific, and powerful alternative therapeutic method for the treatment of refractory focal epilepsy without subjecting intractable patients to toxic doses of medication or irreversible brain damage from epilepsy resections.

## Ethics Statement

This study was carried out in accordance with the recommendations of “principles of animal welfare, refer to animal experiments and experimental animal administrative regulations of Capital Medical University.” The protocol was approved by the “Ethics Committee on Animals Care and Usage of Capital Medical University.” The approval number of IACUC is AEEI-2015-084.

## Author Contributions

XY and DW generated the research idea, study design, and concept. DW, ZY, CJ, WW, JY, and GR performed the experiments and acquired and analyzed the data. XY, DW, FX, and YP drafted the work, made critical revisions for important intellectual content, and interpreted the data. DW wrote the manuscript. XY edited the manuscript. XY, DW, ZY, CJ, WW, JY, GR, FX, and YP approved the final manuscript.

## Conflict of Interest Statement

The authors declare that the research was conducted in the absence of any commercial or financial relationships that could be construed as a potential conflict of interest.
